# Some Interesting Autopsies

**Published:** 1883-10

**Authors:** Frederick Peterson


### Some Interesting Autopsies.

By Frederick Peterson, M. D.

(*Continued from September Number.*)

With regard to these recorded autopsies it is necessary to say that the post mortem examiner has in no case neglected to look at every organ in the body where it was possible to do so. In the majority of cases at charitable institutions there is no difficulty in doing this; but in obductions at private houses the matter is different. These latter generally add nothing to science, and are made as a rule only in the selfish interests of the attending physician, who is anxious to make or to verify his diagnosis. There is only in exceptional instances profit to the pathologist. The post mortem examiner receives usually neither knowledge nor morbid specimens, nor payment for his services. Furthermore, he runs in every case a dangerous risk. In my humble opinion the attending physician in private cases had better make his autopsies himself; for he will then feel responsibility for no life but his own; he will not feel under such deep and lasting obligation to the post mortem examiner (as he otherwise would), and he can always receive satisfactory corroboration of his diagnosis. Where liberal compensation for valuable services is made, the matter is wholly different. Only very rarely do autopsies at private houses add anything to pathological science.

NO. 33—TYPHOID FEVER.

General Hospital, April 30—Frank B., male, æt. 25; dead thirty-six hours; rigor mortis still present; body well built and fairly nourished; sacral bed-sore 5x5 cm., skin, though still covering it, being gangrenous; over seventh cervical vertebra large excoriation; in two or three places on extremities other such excoriations from pressure.

*Head:* Membranes normal; brain itself apparently normal; pineal gland had become a cyst considerably larger than a pea, filled with clear colloid fluid, in which the sand grains were easily felt (*vide* Birch-Hirschfeld's *Pathologische Anatomie*, p. 516), ventricles somewhat enlarged, and ependyma hard.

*Chest:* Left pleura healthy; no adhesions; right pleura had few old adhesions at apex; considerable hypostatic congestion of lungs; purulent bronchitis. Heart very fatty; slight insufficience of aortic valve through union of two of the lunulæ, and there would have been, also, no doubt, a slight stenosis.

*Abdomen:* The so-called colloid degeneration of the recti abdominis very well marked in this case, but not found in the adductors of thigh. On examination under microscope, instead of being homogeneous colloid substance as usual, it was granular fatty degeneration; liver fatty in portions; spleen almost double usual size, flabby and pulpy; kidneys enlarged, pale and fatty; some pyelitis; bladder small, contracted, containing purulent urine; surface raw and covered with ecchymoses (acute cystitis). Peyer's patches ulcerated in ileum, all plaques within the space of eighteen inches from valvula bauhini; also small ulcerations of solitary glands; these ulcers in many places covered with dark blood coagula; fæcal matter yellow; no perforation; no peritonitis.

*Diagnosis:* Cause of death, asthenia from typhoid fever, which led to ileal ulcers, fatty degeneration of heart, liver, kidneys and recti abdominis, pyelitis, cystitis and splenitis.

*Remarks:* The tumor of the pineal gland was rare. In this case it had led to no nervous disturbance. In most recti abdominis of typhoid fever which I have examined, where there has been any degeneration at all, it has been colloid. This is the first in which I have found it fatty degeneration, although it has been recorded before in a few instances.

### NO. 34—KYPHOSIS AND AMYLOID DEGENERATION OF SPLEEN, LIVER, KIDNEYS AND INTESTINES.

Patient of Dr. Storck, May 2—Burkhardt, male, æt. 9; dead twenty-six hours; body emaciated; knees and thighs flexed and inextensible; angular curvature of spine backward at about last dorsal and first lumbar vertebra; old scars in groin from former psoas abscesses; large fistula over sacrum, through which pus poured freely.

Head not examined.

*Chest:* In each pleural cavity about 150 ccm. of sanguinous serum; lungs normal; heart normal.

*Abdomen:* Spleen enlarged, hard, amyloid. Liver enlarged, softer than spleen, both fatty and amyloid. Both kidneys very large, pale and amyloid; posterior surface of right kidney formed part of wall of a psoas abscess, which extended down to ilium and into sacral region. Bodies of first sacral and fifth lumbar vertebræ almost wholly gone, and second and third lumbar also softened. Intestines very pale and amyloid. Stomach normal. Bladder normal.

*Diagnosis:* Spinal caries, causing kyphosis and abscess formation. The long-continued suppuration led to the extensive amyloid degeneration of abdominal viscera described.

*Remarks:* The cause of the caries was a fall from a height five years before.

### NO. 35—MEDULLARY SARCOMA OF PITUITARY BODY.

General Hospital, May 3—Geo. S. Jones, æt. 48; dead six hours; no rigor mortis; body well developed, well formed and well nourished; panniculus adiposus remarkably thick.

*Head:* Brachycephalic. Opacity of pia mater and arachnoid from chronic inflammation; dura mater normal. More than usual quantity of fluid in arachnoid cavity. Where the pituitary body should be was a very soft, pinkish, encapsuled tumor some 5 cm. in diameter, pressing into the brain in front of the pons varolii, compressing the optic commissure, and going deep into the sphenoid bone, having eroded its way into the sphenoidal sinuses. Brain substance around it apparently normal, and the rest of brain substance normal. Under the microscope the suspicious tissue was found to consist of innumerable round cells, of size of white blood corpuscles, lying in alveoli of what appeared to be lymph vessels. Ventricles were very wide, distended with clear serum; ependyma hard; cysts in both choroid plexus.

*Chest:* Costal cartilages ossified. A few old pleuritic adhesions; subpleural ecchymoses. Lungs very œdematous, otherwise normal. Heart fatty degenerated; valves normal; subpericardial ecchymoses; agonia clot in right heart (he was long in dying, by asphyxia); beginning atheroma in aorta.

*Abdomen:* Spleen small, firm in consistence. Liver fatty. Both kidneys somewhat fatty. Bladder distended with urine. Submucosal ecchymoses in stomach and ileum.

*Diagnosis:* Primary medullary sarcoma of pituitary body, followed by chronic meningitis and chronic hydrocephalus internus. Fatty degeneration of heart, liver and kidneys as result of general nutritive disturbance.

*Remarks:* Three years ago this patient began to complain of his eyes failing him. Two years ago his eyes grew worse and he was subject to severe headaches. He had, besides, periods of blindness. His pupils were often much dilated, and often contracted, and always insensitive to light. Up to three days before his death was perfectly intelligent and conscious. The pressure of the tumor upon the optic commissure easily explains the foregoing symptoms. In the last three days he became delirious, then comatose and died. As may be supposed tumors of the pituitaria are exceedingly rare. Disease of this body was once looked upon as a cause of epilepsy—an exploded theory. Colloid degeneration, miliary tubercles and syphilitic gummata have been found in it. Only a few cases of soft sarcoma of the pituitaria are on record.

### NO. 36—ACUTE PRIMARY CARCINOSIS OF LIVER WITH METASTASES IN LUNGS.

Patient of Dr. Bartlett, May 6—Ann Boyne, æt. 44; dead twenty-four hours; body well formed, well nourished, of jaundiced color.

Head not examined.

*Chest:* One litre of dark bloody serum in right pleural sac; one-half litre in left. Right lung much compressed by elevation of diaphragm; both lungs filled with millet seed growths exactly as in subacute tuberculosis, but here and there soft malignant looking nodules of size of hazel nut. Heart atrophied, myocardium fatty, valves normal.

*Abdomen:* Spleen normal in every way. Liver enormously enlarged, weighing eleven pounds, and full of innumerable, soft, white medullary nodules, proved by subsequent microscopic examination to be carcinoma. Besides these, countless hæmotomata existed in the liver, some of which were 5–8 cm. in diameter, evidently formed by ruptured blood vessels in and about the soft cancerous deposits. The gall-bladder was small but not connected with any malignant growth. Pancreas normal in every respect. Both kidneys fatty degenerated, but not abnormal in other respects. Both ovaries and uterus normal. Bladder distended with urine, but normal. Stomach contracted and very small, but normal. Ileum, duodenum, rectum and œsophagus normal.

*Diagnosis:* Acute primary carcinosis of liver with secondary miliary growths in both lungs. Fatty degeneration of heart and kidneys from general nutritive disturbance.

*Remarks:* The woman had been ill but six weeks. Previous to that was healthy and at work. In the family a trace of tuberculosis, but no history of carcinoma or other malignant growth. Such cases are by no means common. According to a verbal statement of von Recklinghausen, with an average of five autopsies daily, he finds but one case of primary carcinoma of the liver every three years. Primary carcinoma of the gall bladder is more common.

### NO. 37—SACCULATED ANEURISM OF AORTA ASCENDENS.

General Hospital, May 9—Geo. Holland, æt. 55, soldier; dead nine hours; body emaciated; soft and yielding tumor 15 cm. in diameter over right mammary region.

*Head:* Venous vessels of brain much engorged. Brain itself normal. No atheromatous disease of basilar arteries.

*Chest:* Œdema of muscular and subcutaneous tissues of thorax. In left pleural cavity nearly a litre of serum. Left lung normal. Pericardium everywhere adherent to heart by connective tissue and gelatinous masses. Heart itself slightly enlarged and somewhat fatty; left ventricle moderately hypertrophied; no valvular lesions; in aorta no atheromatous disease apparent. From aorta ascendens to the right was a large opening capable of admitting four finger breadths; this led into a cavity, bounded by a sac and thrombic layers, large enough to hold the fist; this sac was full of soft recent clots of blood, and protruded near the sternum between the second and third ribs to the distance of 5 cm. beyond the thorax; these two ribs were crowded apart and eroded, the second on its lower border, the third on its upper, so that these ribs were there but 5 mm. in width; on the left side of this part of the sac, external to the thorax, was an opening which would pass a finger, where rupture had evidently occurred some time previously; through this the blood had gushed out into the tissues, forming, external to the thorax, a diffuse arterial hæmatoma around the true aneurism, so that this extra-thoracic tumor, a few days before death, measured 25 cm. in diameter (this, of course, was less post mortem because of the rupture internally); the aneurism itself had made its way behind and to the right into the greater part of the middle and lower lobes of the right lung, and there the tumor protruded from the thorax; the pleural surfaces had grown fast together; there was no evidence of pleurisy elsewhere; at the lowest part of the lung was a small tear in the aneurismal sac, 2½ cm. in length, opening into the pleural cavity; the pleural cavity contained fully two litres of recent blood coagula, with a little serum. The fatal rupture had taken place here the day before. Between the third and fourth ribs was also a large space, the fourth being pressed downwards and much eroded. The arca aortæ and aorta descendens normal.

*Abdomen:* Spleen normal. Liver fatty. Kidneys, stomach ileum and bladder normal. Small quantity of serum in abdominal cavity.

*Diagnosis:* Sacculated aneurism of aorta ascendens. Rupture of this caused death.

*Remarks:* For some time a proper history of the case could not be elicited from the patient, hence he was the object of great interest to a number of the physicians of the city, and the diagnoses varied considerably. There was, however, no difficulty in making a proper diagnosis, as soon as it was learned that the patient had for years had a pulsating tumor of small size in the right mammary region, which suddenly, within a few months, began to enlarge swiftly. His sudden death was also corroborative. Through the kindly assistance and advice of a dozen or more of the physicians present at the autopsy I was enabled to remove the specimen entire, and it is preserved in the college museum.

NO. 42—ANOTHER SACCULATED AORTIC ANEURISM.

Patient of Dr. Rochester, May 23d — Zhach, male, æt. 39; dead twenty-seven hours. Body extremely emaciated.

Head not examined.

*Chest:* Right lung normal; no adhesions; left lung everywhere firmly attached to the thorax; upon cutting into it, found it filled with innumerable large and small abscesses, from size of pea to that of hazel nut and larger; bronchial tubes also full of pus; no tubercles. Pericardium contained over one litre of serum full of lymph flocculi; both parietal and visceral pericardium covered with rough, villous fibrin. Heart and valves normal. Atheromatous disease of aorta in first stages. At juncture of descending aorta and arch an opening large enough to admit a finger into an aneurism twelve cm. in diameter. This tumor had eroded the bodies of the seventh cervical and first and second dorsal vertebræ, their left transverse processes, and also part of corresponding left ribs. The rest of the thoracic and the abdominal aorta normal.

*Abdomen:* Spleen slightly enlarged and hard. Liver engorged. Both kidneys somewhat atrophied, fatty, and contained a few scars of old cysts.

*Diagnosis:* Sacculated aortic aneurism. This caused suppurative pneumonia of left lung and pericarditis with effusior. Death from asthenia.

NO. 62—NON-SACCULATED ANEURISM OF AORTA DESCENDENS.

Sisters of Charity Hospital, Aug. 24—Michael Delaney, æt. 47; dead eighteen hours; body well nourished; very marked rigor mortis; blood oozing from mouth.

Head not examined.

*Chest:* A few old adhesions of right lung; left adherent to ribs behind; some œdema of right apex, and hypostatic congestion of lowest lobe. Pericardium very thin with old pericarditis flecks on visceral portion. Heart enlarged and fatty; no blood whatever in heart or venæ cavæ; aortic valves somewhat thickened; aortic arch dilated and atheromatous; an aneurism 12 cm. in diameter of descending aorta, just behind heart; this was an aneurismal dilation of the vessel or a non-sacculated aneurism; it was full of coagula; behind it had eroded a great part of the bodies of three or four dorsal vertebræ, and had pushed into the left lung until its sac touched the ribs behind; there were numerous small abscesses in the lung close to the aneurismal sac; the œsophagus was full of dark blood coagula, and there was a small opening large enough to pass a lead pencil in its posterior wall where the aneurism had ruptured; there was some infiltration of subpleural cellular tissue with blood.

*Abdomen:* Stomach enormously dilated, extending to umbilicus, containing one large coagulum of blood accurately moulded to its shape; blood also in duodenum. Spleen enlarged and soft. Liver small and very fatty. Both kidneys fatty, containing a few cysts each, atrophied and granular. Lime plates in aorta at iliac bifurcation.

*Diagnosis:* Aneurismal dilation of aorta descendens. Rupture into œsophagus the immediate cause of death. General endarteritis deformans was the cause of the aneurism. Chronic interstitial nephritis.

NO. 17—INTERNAL HEMORRHAGE FROM RUPTURE OF A SMALL ARTERY IN ENDARTERITIS DEFORMANS.

Patient of Dr. Burwell, Jan. 20—Mrs. S., æt. 68; dead twenty hours; body very well nourished; rigor mortis still present.

Brain not examined.

Chest not examined.

*Abdomen:* In abdominal cavity some 200 ccm. of blood coagula. Spleen small, elongated, black and hard, and vena lienalis very large. Peculiar soft blotches on mesentery not elevated, neither tubercular nor cancerous. Liver atrophied and exceedingly fatty, and had two rib marks from corset atrophy. Omentum and mesentery very fat. About eighty gall stones of cholesterin and bile pigment, size of pepper grains, in gall-bladder. In stomach great capillary congestion, even ecchymoses, but no mucus. Intestines normal; no peritonitis. Great fatty degeneration and atheroma of arteries, in some places atheromatous ulcers and extravasations into media. Kidneys: pyelitis, and granular atrophy, with cysts. Bladder, uterus and ovaries normal. The point of bleeding was in one of the smaller branches of the splenic artery.

*Diagnosis:* Cause of death, rupture of some branch of splenic artery, with hemorrhage into peritoneal cavity and death from shock. Chronic interstitial nephritis.

*Remarks:* The woman died suddenly with collapse. The general atheromatous disease of the arteries had so weakened the walls of those vessels that they were ready, at any slight increase in blood pressure, or by any sudden turn of the body, to burst. The following case is so similar that I place the two in juxtaposition for comparison.

NO. 21—ABDOMINAL HEMORRHAGE. ATHEROMATOUS DISEASE OF ARTERIES.

Patient of Dr. Harrington, Feb. 6—Mr. S., æt. 53; dead fourteen hours. Body well nourished.

Head not examined.

*Chest:* A few subpleural ecchymoses. Lungs both normal. Heart and valves normal. No atheromatous disease in thoracic aorta.

*Abdomen:* Fully four litres of blood in the peritoneal cavity. Spleen slightly atrophied. Liver also small and fatty. Kidneys small but normal. A slightly marked chronic gastritis. Rupture of one of the smallest pancreatic branches of the splenic artery. There was general atheromatous disease of the abdominal aorta and its branches, in many places large calcareous plates in the vascular walls.

*Diagnosis:* Death from shock caused by hemorrhage into abdominal cavity, from rupture of an atheromatous artery.

*Remarks:* It is generally stated in works on pathological anatomy that the arch of the aorta is most usually affected when atheromatous disease exists at all. In this case it was only to be found in the abdominal aorta. I would not mention this if I had not found it to be a fact, in at least twenty cases, that where any atheromatous disease exists it is certain to be at the bifurcation of the iliacs, although the thoracic aorta may not be affected at all, or to a very slight extent.

(*To be continued in next number of the Journal.*)

